# Dynamic monitoring and analysis of factors influencing ecological environment quality in northern Anhui, China, based on the Google Earth Engine

**DOI:** 10.1038/s41598-022-24413-0

**Published:** 2022-11-24

**Authors:** Xia Wang, Xiaojie Yao, Changzheng Jiang, Wei Duan

**Affiliations:** School of Architecture and Planning, Anhui Jianzhu University, Hefei, 230022 China

**Keywords:** Ecology, Environmental sciences

## Abstract

Monitoring the ecological environment quality is an important task that is often connected to achieving sustainable development. Timely and accurate monitoring can provide a scientific basis for regional land use planning and environmental protection. Based on the Google Earth Engine platform coupled with the greenness, humidity, heat, and dryness identified in remote sensing imagery, this paper constructed a remote sensing ecological index (RSEI) covering northern Anhui and quantitatively analyzed the characteristics of the spatiotemporal changes in the ecological environment quality from 2001 to 2020. Geodetector software was used to explore the mechanism driving the characteristics of spatial differentiation in the ecological environment quality. The main conclusions were as follows. First, the ecological environment quality in northern Anhui declined rapidly from 2001 to 2005, but the rate of decline slowed from 2005 to 2020 and a trend of improvement gradually emerged. The ecological environment quality of Huainan from 2001 to 2020 was better and more stable compared with other regional cities. Bengbu and Suzhou showed a trend of initially declining and then improving. Huaibei, Fuyang, and Bozhou demonstrated a trend of a fluctuating decline over time. Second, vegetation coverage was the main influencing factor of the RSEI, while rainfall was a secondary factor in northern Anhui from 2001 to 2020. Finally, interactions were observed between the factors, and the explanatory power of these factors increased significantly after the interaction. The most apparent interaction was between vegetation coverage and rainfall (*q* = 0.404). In addition, we found that vegetation abundance had a positive impact on ecological environment quality, while population density and urbanization had negative impacts, and the ecological environment quality of wetlands was the highest. Our research will provide a theoretical basis for environmental protection and support the high-quality development of northern Anhui.

## Introduction

The environment is the sum of various environmental factors related to both ecosystems and human activities, and it has a profound impact on the natural and social environment of humans^1^. With the rapid development of urbanization and the increasing intensity of human activities, a growing number of cities face environmental problems, such as the urban heat island effect, water shortages, and air pollution^2–4^. While the international community continues to strengthen the protection and governance of the environment, a timely assessment of the temporal and spatial changes in ecological environmental quality is very important for environmental protection and sustainable regional development. In 2006, the *Technical Criterion for Ecosystem Status Evaluation* issued by the *Ministry of Environmental Protection of China* introduced an ecological index (EI) composed of indices of biological abundance, water network density, vegetation cover, and environmental quality^5^ and conducted an annual comprehensive evaluation of the current situation and development trend of China’s regional environment, which has been widely used in the evaluation of regional environments above the county level. However, in practical applications, this EI also faces many problems, including difficulty in obtaining indicator data and low data accuracy. Therefore, developing a more comprehensive, convenient, and accurate method that can be used to evaluate the ecological environment quality is of great significance to the long-term dynamic monitoring, management, and sustainable social development of the environment.

Remote sensing images are convenient to acquire and provide a short return interval and a long time sequence. These images have become an important data source for evaluating ecological environmental quality. At present, scholars have developed a variety of remote sensing indices to describe ecological situations that have been applied to forests^6–8^, wetlands^9–12^, grasslands^13^, farmlands^14–16^, and lakes^17^. However, these indices only evaluate one aspect of the environment and often reflect one-sided ecological conditions. For example, the normalized difference vegetation index (NDVI) and normalized difference built-up index (NDBI) are used to reflect the growth status of plants in many study areas^18–20^, as well as the distribution of urbanized land^21,22^, while the land surface temperature (LST) has been used to study the effects of the urban thermal environment^23–25^. To cope with these challenges, Xu proposed a remote sensing ecological index (RSEI) that integrates the four indicators of greenness, dryness, humidity, and heat and extracts a single variable that can represent multiple variables without artificially setting the weight^26^. This method can conveniently and objectively model and predict the conditions in the environment of a study area and analyze its temporal and spatial evolution^5,27^.

To date, RSEI has been applied in areas including nature reserves^28^, urban agglomerations^29,30^, archipelagos^31^, and watersheds^32–34^. Some scholars have combined R language to conduct Mantel and Pearson correlation analyses on the RSEI, NDVI, TC-Wetness (Wet), normalized difference building-soil index (NDBSI), LST, precipitation, land use, and gross domestic product (GDP) in a given study area. The results show that NDVI is the key factor controlling the quality of the RSEI score^35^. In addition, scholars have used spatial autocorrelation to explore the spatial correlation between ecological quality and ecosystem services in the southern red soil hilly watershed of China. The results show that the spatial distribution of the two factors is similar, and among all ecosystem services, carbon storage has the highest correlation with RSEI^36^. Many studies have shown that RSEI is conducive to the quantitative evaluation of changes in the ecological environment quality on a regional scale^37,38^. Although RSEI has been widely used, meteorological problems, such as the amount of cloud cover in remote sensing images, which varies in different periods, have created challenges in the use of remote sensing data. It is difficult to remove clouds when the amount of cloud cover is large, and image stitching using images obtained during different periods is also difficult. The Google Earth Engine (GEE), launched by Google in 2010, is a visual cloud computing platform that integrates massive quantities of remote sensing data and multiple processing methods. It can solve problems such as those related to cloud removal, multi-period image stitching, and cumbersome processing caused by large areas. The processing time is greatly reduced, and the calculation work can be completed efficiently. Based on this advantage, the GEE has been widely used in land-monitoring efforts, including land cover classification and dynamic monitoring^39,40^, geographic mapping^41–43^, and change monitoring^44–46^.

Research on the factors affecting ecological environment quality can explore effective ways to improve this environment. Yin et al. used Pearson correlation analysis to determine the correlation between ecological environment quality and several variables^47^. Nie et al. implemented spatial autocorrelation to explore the spatial differentiation of the coal mine ecological index (CMEI) ^48^. Li et al. applied ordinary least squares(OLS) and geographically weighted regression (GWR) models to explore the relationship between the spatial distribution of soil heavy metal increments and environmental factors^49^. Most of the above research methods use spatial econometric models. Although based on the perspective of spatial correlation, this global model is easy to cover up the heterogeneity of research objects when it is used to analyze objects with heterogeneity, thus leading to erroneous results caused by mixed effect interference^50^. In addition, these models are used to analyze the single indicator of ecological environment impact, but ignore the possible interaction between different influencing factors. Therefore, it is necessary to use more specialized tools to analyze heterogeneous objects. Geodetector is a statistical method that Wang proposed to detect spatial differentiation and reveal its driving factors, which can quantitatively describe the relative importance of the interactions of influencing factors. Therefore, we can apply the Geodetector model to analyze the social and natural factors that affect ecological environment quality.

Through the above analysis, it is found that using the RSEI model to analyze the ecological environment quality in the study area has become the mainstream trend. However, the existing literature has faced various challenges in the processing of remote sensing images, the quantitative analysis of the impact of each variable on RSEI is not perfect, and the impact of each variable on environmental quality has not been well explained so far. To make up for the shortcomings of the existing research, this paper selected northern Anhui as the research object, efficiently constructed an RSEI based on the GEE platform by integrating moderate resolution imaging spectroradiometer (MODIS) satellite image data, quantitatively analyzed the temporal and spatial characteristics of the ecological environment quality in northern Anhui from 2001 to 2020, and through the Geodetector explored the driving mechanisms of the spatial differentiation of RSEI in northern Anhui from 2001 to 2020.

This paper is innovative due to the following two aspects. First of all, based on GEE's powerful cloud computing capability, we established a time threshold to ensure the consistency of the time periods studied and used cloud removal and image median equalization to improve the original image quality. Secondly, we used Geodetector to more comprehensively analyze the influencing factors of ecological environment quality in northern Anhui, including determining the importance of each factor on ecological environment quality, the interactions between the influencing factors, and the influential trend of each factor on ecological environment quality.

The rest of this paper is arranged as follows. The second part introduced the basic situation of the study area, as well as the data sources and analysis methods used in this paper. The third part constructed the RSEI and analyzed the space–time characteristics of ecological environment quality in northern Anhui. The fourth part explored the influencing factors of spatial differentiation of RSEI in northern Anhui. The fifth part discussed the main findings and drew several conclusions.

## Study area

**Figure 1:**
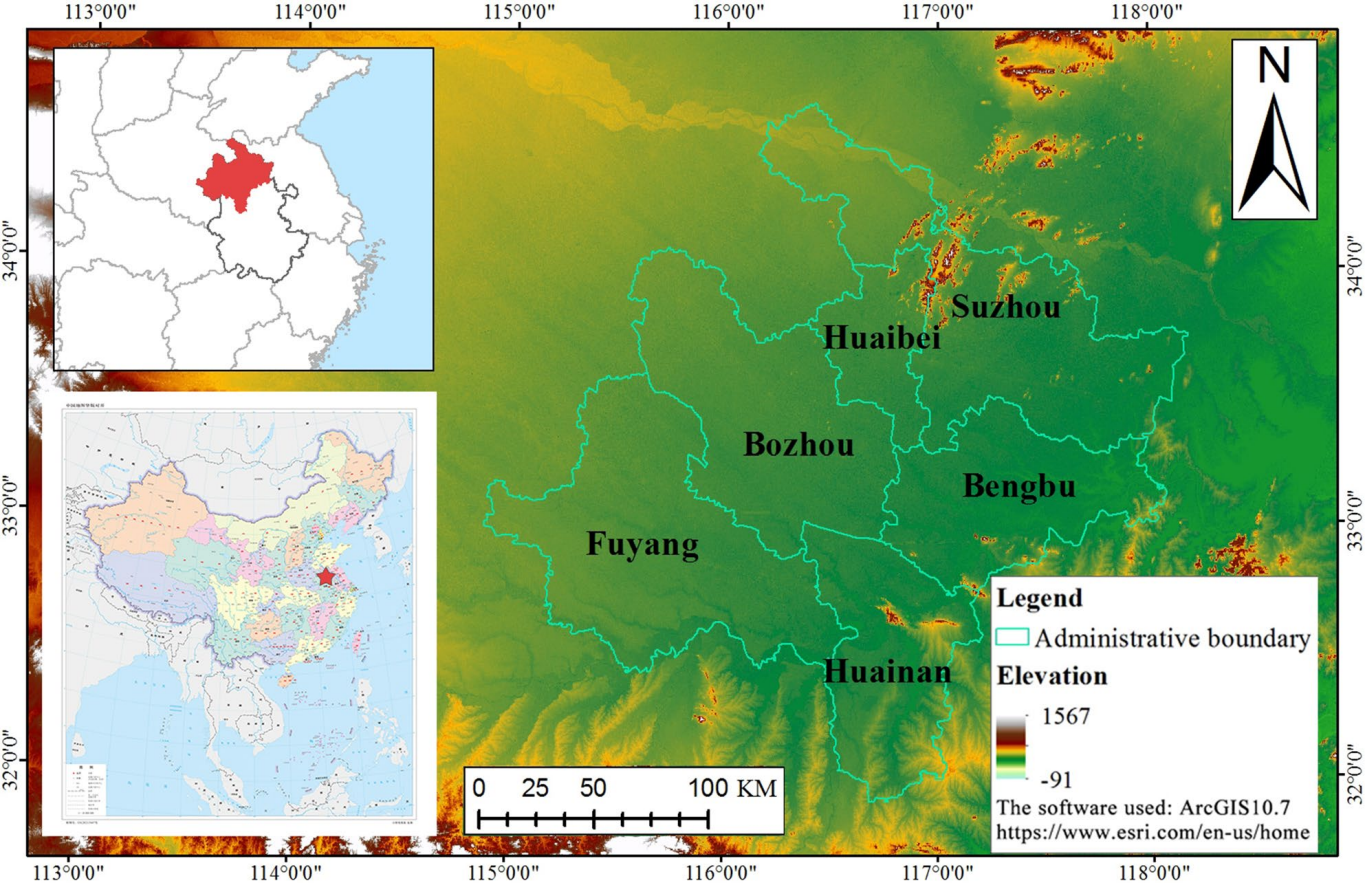
Schematic map of the study area.

Northern Anhui Province, China (Fig. 1) consists of six cities: Suzhou, Huaibei, Bengbu, Fuyang, Huainan, and Bozhou. The flat terrain of northern Anhui slopes slightly from the northwest to the southeast. Northern Anhui has a warm temperate semi-humid monsoon climate with obvious monsoons, a mild climate, and moderate precipitation. Water resources in northern Anhui are abundant and mainly consist of natural precipitation, river water, and groundwater. The average annual precipitation in the entire region is 820–950 mm, and the average annual runoff of river water is 3.822 billion m^3^. The entire area of the northern Anhui Province is relatively flat, which provides excellent farming conditions for agricultural production, making northern Anhui an important grain production area in the province. In 2018, the total sown area of crops in northern Anhui reached 4.66 million hm^2^, accounting for 53.14% of the province. However, the economic development of northern Anhui has lagged for a long period. According to statistics, in 2018, except in Bengbu, the per capita GDPs of the other five cities were lower than the average level of the province, making them the most underdeveloped areas in the province. Northern Anhui has a large population and extensive natural resources. In the context of China’s rapid economic development and urbanization, it has attained numerous achievements. However, as northern Anhui has developed, it has consistently relied on resource consumption to promote economic development and has sacrificed its ecological environment to expand its urban boundaries, resulting in resource shortages and environmental pollution, which has restricted its subsequent development momentum. In recent years, China has vigorously promoted the economic development of the Yangtze River Delta and emphasized the need to solve the problem of unbalanced development. Northern Anhui is an underdeveloped region in the Yangtze River Delta. As an inland hinterland and major agricultural production area, it has a large working population. These elements constitute its basic guarantee to promote its high-quality development. We can rely on the overall planning of land and space, maximize regional advantages and policy conditions, and promote this region’s high-quality development. Therefore, an in-depth exploration of the characteristics and influencing factors of the evolution of ecological environment quality in northern Anhui can provide valuable references for the rational use of land resources and the promotion of an ecological civilization.

## Data collection

The data used in this paper are shown in Table 1, our study years are 2001, 2005, 2010, 2015, and 2020. The administrative boundary data were obtained from the China Geographic Information Resource Directory Service System. The MODIS satellite product was obtained from the United States Geological Survey. Among the MODIS dataset, the MOD13A1 V6 dataset contains NDVI and the enhanced vegetation index, with a resolution of 500 m, and synthesis using the best pixels available within a 16-day period. The MOD11A2 V6 dataset contains daytime and nighttime surface temperatures. Its synthesis period is 8 days, and the resolution is 1000 m. The MOD09A1 V6 dataset provides seven reflectance bands, all of which have undergone atmospheric correction to remove problematic data, such as that caused by atmospheric and aerosol scattering. We used the quality control band to perform cloud and cloud shadow removal preprocessing on the surface reflectance data acquired to obtain a high-quality time series of surface reflectance dataset. In addition, the modified normalized difference water index was used to mask the water of each indicator in the study area to eliminate the influence of water on humidity. The temperature and rainfall data were obtained from the National Earth System Science Data Center, National Science & Technology Infrastructure of China. The temperature was the average temperature, and the rainfall was the cumulative rainfall^51^. The vegetation coverage values were obtained from the NDVI layer of the MOD13A1 V6 dataset using the maximum value synthesis method and the binary pixel model, with a spatial resolution of 500 m. The nighttime light data were obtained from the Earth Observation Group, with a spatial resolution of 1000 or 500 m. The land use data were obtained from the MCD12Q1 V6 dataset, with a spatial resolution of 500 m. The population density data were obtained from WorldPop, with a spatial resolution of 1000 m. All data were uniformly sampled to an accuracy of 500 m and projected to the WGS_1984_UTM_Zone_50N coordinate system to ensure the consistency of the data. For data that were more sensitive to seasonal factors, such as MODIS satellite products, the temperature, rainfall, and vegetation coverage were chosen from June to September (summer) every year to eliminate the interference of the different seasons on the research results.

**Table 1:** Data information.

Data name	Data description	Time	Data format/accuracy	Data source
Administrative boundary	Official government planning boundary	2017	Shp	https://www.webmap.cn
MOD13A1 V6	Remote sensing satellite data	June to September in 2001, 2005, 2010, 2015 and 2020	Raster/500 m	https://www.usgs.gov/
MOD11A2 V6			Raster/1000 m	https://www.usgs.gov/
MOD09A1 V6			Raster/500 m	https://www.usgs.gov/
Temperature	Meteorological data		Raster/1000 m	http://www.geodata.cn/
Rainfall			Raster/1000 m	http://www.geodata.cn/
Vegetation coverage	It reflects the abundance of vegetation		Raster/500 m	Calculated by NDVI data in the MOD13A1 V6 dataset
Land use	It reflects the nature and use of land use units	2001, 2005, 2010, 2015 and 2020	Raster/500 m	https://www.usgs.gov/
Nighttime light	It reflects the degree of economic development		Raster1000 m/500 m	https://ngdc.noaa.gov/ https://eogdata.mines.edu/products/vnl/
Population density	It reflects the distribution of the population		Raster/1000 m	www.worldpop.org

## Research methods

**Figure 2:**
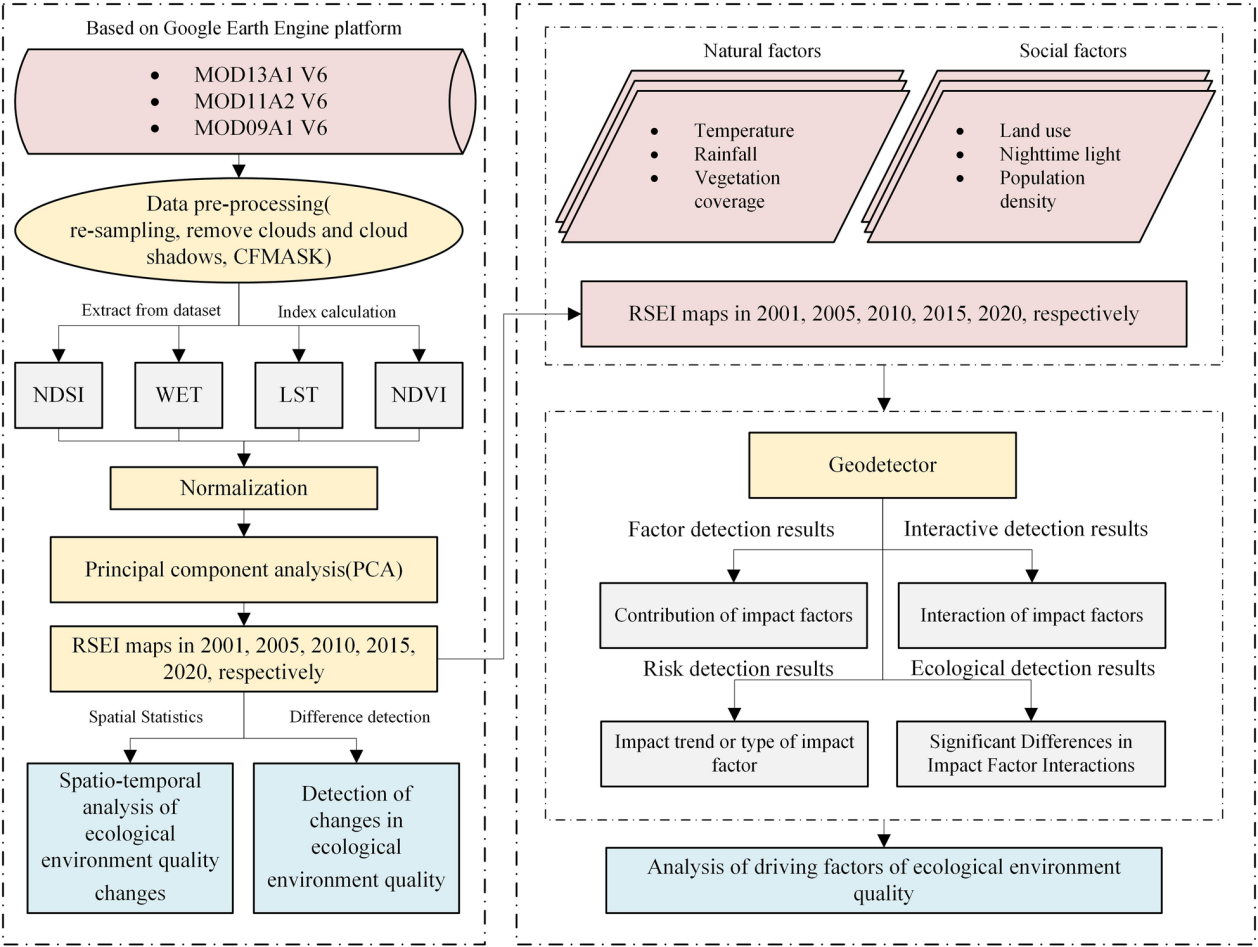
Workflow.

Figure 2 shows the research workflow. First, the MODIS dataset based on the GEE was obtained, and RSEI maps were created for June to September of 2001, 2005, 2010, 2015, and 2020. Second, five RSEI maps from 2001 to 2020 were used to analyze the characteristics of the temporal and spatial changes in the ecological environment quality in northern Anhui. Finally, the influencing factors of ecological environment quality in the study area were explored using Geodetector.

### Building the RSEI

Principal component analysis (PCA) is an evaluation method based on dimension reduction. Its connotation is to analyze and sort multiple evaluation indicators, and then extract one or several representative indicators to replace the overall evaluation indicators. The extracted representative indicators are the so-called principal components. The advantage of this index extraction method is that it can effectively simplify the problem, use fewer indicators to evaluate the whole system, reduce the amount of calculation, and will not lose the effective information in the overall indicators. Through PCA, RSEI is calculated by integrating four indicators of greenness, dryness, humidity, and heat. First, use the following formulas for normalization:

1 $$y_{i} = \frac{{x_{i} - \overline{x}}}{s}\quad (i = 1,2, \ldots, n),$$

2 $$\overline{x} = \frac{1}{n}\sum\limits_{i = 1}^{n} {x_{i} } ;\quad s = \frac{1}{n - 1}\sum\limits_{i = 1}^{n} {(x_{i} - \overline{x})^{2} ,}$$

where *n* is the number of indicators; *x*_*i*_ is the original value of the indicator; $\overline{x}$ is the mean value; *s* is the standard deviation; *y*_*i*_ is the normalized value. Based on the standardized data, the covariance matrix of each variable is established, as follows:

3 $$R={({r}_{i,j})}_{p*p,}$$

where *R* is the covariance matrix formed by the mean value of each eigenvalue data; *i* and *j* represent the *i*th feature and the *j*th feature, respectively, and ${r}_{i,j}$ is their covariance; *p* is the original total characteristic number. Then, the eigenroot ${\lambda }_{1}$≥${\lambda }_{2}$≥…≥${\lambda }_{p}$ of the matrix *R* is calculated and the corresponding unit eigenvector ${\alpha }_{1},{ \alpha }_{2}$, …,${\alpha }_{p}$,

4 $${\alpha }_{1}=\left[\begin{array}{l}{\delta }_{11}\\ {\delta }_{21}\\ \dots \\ {\delta }_{p1}\end{array}\right],{\alpha }_{2}=\left[\begin{array}{l}{\delta }_{12}\\ {\delta }_{22}\\ \dots \\ {\delta }_{p2}\end{array}\right],\dots ,{ \alpha }_{p}=\left[\begin{array}{l}{\delta }_{1p}\\ {\delta }_{2p}\\ \dots \\ {\delta }_{pp}\end{array}\right],$$

where $\delta$ is the value of a direction component of an eigenvector after solving the covariance matrix. A linear combination of the eigenvectors is then made, as in formula (5),

5 $${F}_{i}={\alpha }_{1i}{X}_{1}+{\alpha }_{2i}{X}_{2}+\dots +{{\alpha }_{pp}X}_{p} (i={1,2},\dots ,p),$$

After outputting *m* principal components, the comprehensive index *Y* is calculated,

6 $$Y={\alpha }_{1}{Y}_{1}+{\alpha }_{2}{Y}_{2}+\cdots +{\alpha }_{m}{Y}_{m,}$$

where ${Y}_{i}$ is the *i*th principal component, and ${\alpha }_{i}$ is the *i*th eigenvector. The calculation method is shown in formula (7),

7 $${\alpha }_{i}={\lambda }_{i}/\sum_{i=1}^{m}{\lambda }_{i},$$

where NDVI represents the greenness index, using the NDVI layer in the MOD13A1 V6 dataset. The LST represents the heat index, using the daytime surface temperature layer in the MOD11A2 V6 dataset. In addition, NDBSI stands for a dryness index, which is synthesized using a soil index (SI) and a building index (IBI) ^52^. Wet stands for the humidity index, which is the main component data for humidity obtained through the tasseled cap transformation of MODIS data^34^. These data were calculated using each band in the MOD09A1 V6 dataset. The index formula is as follows:

8 $$Wet=0.1147\,Red\,+\,0.2489\,NIR1\,+\,0.2408\,Blue\,+\,0.3132\,Green\,-\,0.3122\,NIR2\,-\,0.6416\,SWIR1\,-\,0.5087\,SWIR2,$$

9 $$NDBSI=\frac{SI\,+\,IBI}{2},$$

10 $$SI=\frac{\left(SWIR1\,+\,Red\right)-\left(NIR\,+\,Blue\right)}{\left(SWIR1\,+\,Red\right)+\left(NIR\,+\,Blue\right)},$$

11 $$IBI=\frac{\frac{2SWIR1}{SWIR1\,+\,NIR}-\left[\frac{NIR}{NIR\,+\,Red}+\frac{Green}{Green\,+\,SWIR1}\right]}{\frac{2SWIR1}{SWIR1\,+\,NIR}+\left[\frac{NIR}{NIR\,+\,Red}\,+\,\frac{Green}{Green\,+\,SWIR1}\right]},$$

where Red, Near Infrared 1 (NIR1), Blue, Green, Near Infrared 2 (NIR2), shortwave infrared 1 (SWIR1), and shortwave infrared 2 (SWIR2) represent bands 1–7 of the MOD09A1 surface reflectance products, respectively.

#### Geodetector

Geodetector is a statistical method used to detect spatial differentiation and reveal the driving factors and mechanisms influencing spatial differentiation. This method analyzes the explanatory power and contribution of the influencing factors by comparing the consistency of the spatial distribution of the target factor and the influencing factor^50^. The core idea is as follows: if a certain independent variable has an important influence on the dependent variable, then the spatial distributions of the independent and dependent variables are similar. Geodetector includes four types of detectors: factor, interaction, risk, and ecological detectors.

The factor detector mainly uses the explanatory power of the factor to quantitatively measure the degree of influence of each natural factor on the spatial differentiation of RSEI in northern Anhui. Among them, the expression formula of the explanatory power *q* is:

12 $$q=1-\frac{\sum_{h=1}^{L}{N}_{h}{\sigma }_{h}^{2}}{N{\sigma }^{2}},$$

where *h* = 1,…, L is the stratification number of the influencing factor; *N*_*h*_ and *N* represent the sample number of the influencing factor in the layer *h* and the entire region of northern Anhui, respectively; and σ_h_^2^ and σ^2^ represent the variance of the RSEI value of the impact factor in layer *h* and the entire region of northern Anhui, respectively. The value range of *q* is [0, 1], where the larger the value of *q*, the stronger the explanatory power of the influencing factor on the spatial distribution of RSEI in northern Anhui. In addition, *q* = 0 means that the factor has no relationship with the RSEI, while *q* = 1 means that the factor can fully explain the spatial distribution of the RSEI.

The interaction detector is used to detect whether the interaction between two influencing factors will increase or decrease the explanatory power of the RSEI, or whether the effects of these factors on the RSEI are independent of each other. The judgment basis is shown in Table 2.

**Table 2:** Types of interaction between influencing factors.

Judgments based	Type of interaction
*q*(*X1* ∩ *X2*) < Min(*q*(*X1*), *q*(*X2*))	Non-linear reduction
Min(*q*(*X1*), *q*(*X2*)) < *q*(*X1* ∩ *X2*) < Max(*q*(*X1*), *q*(*X2*))	Single-factor non-linear reduction
*q*(*X1* ∩* X2*) > Max(*q*(*X1*), *q*(*X2*))	Two-factor enhancement
*q*(*X1 *∩ *X2*) = *q*(*X1*) + *q*(*X2*)	Independent
*q*(*X1* ∩ *X2*) > *q*(*X1*) + *q*(*X2*)	Non-linear enhancement

The risk detector is used to determine whether there is a significant difference between the mean values of an attribute of the two factors between the sub-regions and is used to search for regions with high mean RSEI values. The detector uses *t* to test this:

13 $${t}_{{\overline {y}}_{h=1}-{\overline{y}}_{h=2}}=\frac{{\overline{Y}}_{h=1}-{\overline{Y}}_{h=2}}{{\bigg[\frac{{\text{Var}}({\overline{Y}}_{h=1})}{{n}_{h=1}}+\frac{{\text{Var}}({\overline{Y}}_{h=2})}{{n}_{h=2}}\bigg]}^{1/2}},$$

where ${t}_{{\overline{y}}_{h=1}-{\overline{y}}_{h=2}}$ represents the difference between two subregions *h* = *1* and *h* = *2*; $\overline{Y}$_*h*_ represents the mean value of the attributes in subregion *h*; *n*_*h*_ is the number of samples in subregion *h*; Var represents the variance.

The ecological detector is used to determine whether there is a significant difference between the effects of two influencing factors, *X1* and *X2*, on the spatial distribution of the RSEI in northern Anhui, as measured by *F*:

14 $$F=\frac{{N}_{X1}\times ({N}_{X2}-1)\times {\text{SSW}}_{X1}}{{N}_{X2}\times ({N}_{X1}-1)\times {\text{SSW}}_{X2}},$$

15 $${\text{SSW}}_{X1}=\sum_{h=1}^{L1}{N}_{h}{\sigma }_{h}^{2},{\text{SSW}}_{X2}=\sum_{h=1}^{L2}{N}_{h}{\sigma }_{h}^{2},$$

where *N*_*X1*_ and *N*_*X2*_ represent the number of samples of impact factors *X1* and *X2*, respectively; *L1* and *L2* represent the number of layers of impact factors *X1* and *X2*, respectively; *SSW*_*X1*_ and *SSW*_*X2*_ represent the sum of the intra-layer variance of impact factors *X1* and *X2*, respectively.

## Results

### PCA results

Before the principal component analysis, we conducted the Kaiser–Meyer–Olkin (KMO) and Bartlett's sphericity test on the four indicators constituting RSEI in 2001, 2005, 2010, 2015, and 2020 respectively. The KMO values were 0.731, 0.653, 0.580, 0.785, and 0.649, respectively, which were greater than 0.5. At the same time, the significance values in Bartlett's sphericity test were all 0.000. The results showed that these data were suitable for PCA. As shown in Table 3, the contribution rates of the first principal component (PC1) in the three periods of 2001, 2005, and 2010 were all higher than 60%, and the contribution rates in 2015 and 2020 exceeded 70%. Compared with PC1, the contribution rate of other principal components was lower than 30%, indicating that PC1 integrated most of the characteristics of the four ecological indicators. The load values of the humidity index and the greenness index in PC1 of the 5 years were positive, while the load values of the dryness index and the heat index were both negative. This showed that the humidity and greenness indicators had a positive effect on the regional ecological environment, while the dryness and heat indicators had a negative impact. This is in line with the actual situation. The load values of each index in the other principal components were unstable. For example, the load value of the heat index in the second principal component (PC2) in 2001 was positive, while the load value of the humidity index was negative, indicating that heat had a positive impact on the ecological environment, while humidity had a negative impact on the ecological environment, which is obviously inconsistent with the actual situation. At present, the ecological meaning of this phenomenon was difficult to explain. Compared with other components, PC1 had obvious advantages. It could integrate the characteristic information of four indicators and be consistent with the actual situation. Therefore, it can be used to establish a comprehensive ecological index and analyze the changes in the ecological environment in the study area.

**Table 3:** PCA results.

Year	Index	PC1	PC2	PC3	PC4
2001	NDVI	0.565	0.521	0.072	−0.636
	LST	−0.339	0.091	0.928	−0.122
	WET	0.239	−0.845	0.108	−0.467
	NDBSI	−0.713	0.086	−0.348	−0.602
	Eigenvalues	0.034	0.01	0.007	0.001
	Contribution rate	65.4%	19.2%	13.5%	1.9%
2005	NDVI	0.499	−0.779	−0.309	−0.219
	LST	−0.294	−0.179	−0.558	0.755
	WET	0.550	0.601	−0.576	−0.070
	NDBSI	−0.602	−0.010	−0.511	−0.614
	Eigenvalues	0.016	0.006	0.002	0.002
	Contribution rate	61.5%	23.1%	7.7%	7.7%
2010	NDVI	0.408	0.560	0.350	0.630
	LST	−0.234	−0.224	0.931	−0.167
	WET	0.408	−0.796	−0.008	0.447
	NDBSI	−0.783	−0.057	−0.100	0.612
	Eigenvalues	0.019	0.009	0.002	0.001
	Contribution rate	61.3%	29.0%	6.5%	3.2%
2015	NDVI	0.545	0.612	−0.179	−0.545
	LST	−0.304	0.545	0.779	0.051
	WET	0.199	−0.569	0.515	−0.609
	NDBSI	−0.756	0.072	−0.308	−0.574
	Eigenvalues	0.036	0.003	0.002	0.001
	Contribution rate	85.7%	7.1%	4.8%	2.4%
2020	NDVI	0.587	0.650	−0.055	0.479
	LST	−0.336	0.350	0.874	0.037
	WET	0.345	−0.672	0.380	0.534
	NDBSI	−0.651	0.050	−0.299	0.696
	Eigenvalues	0.028	0.006	0.003	0.001
	Contribution rate	73.7%	15.8%	7.9%	2.6%

The load values of each component index of PC1 showed that the greenness index first decreased and then increased, reaching the lowest value of 0.408 in 2010, indicating that the vegetation coverage in northern Anhui has gradually improved since 2010. The humidity index showed a trend of first rising and then fluctuating and falling, reaching the highest value of 0.550 in 2005, indicating that the influence of soil moisture on the ecological environment gradually weakened after 2005. The absolute value of the dryness index showed a trend of rising first and then falling, and reached the highest value of 0.783 in 2010, indicating that from 2001 to 2010, human activities led to a significant expansion of the urban area, but after 2010, it has been basically controlled. The heat index can usually reflect the ecological disturbance caused by the heat island effect caused by human activities. Its absolute value showed a trend of first decreasing and then increasing. Its lowest value was 0.234 in 2010, indicating that the heat island effect in the study area has increased year by year since 2010.

### Temporal and spatial analysis of the ecological environment quality change in northern Anhui

**Figure 3:**
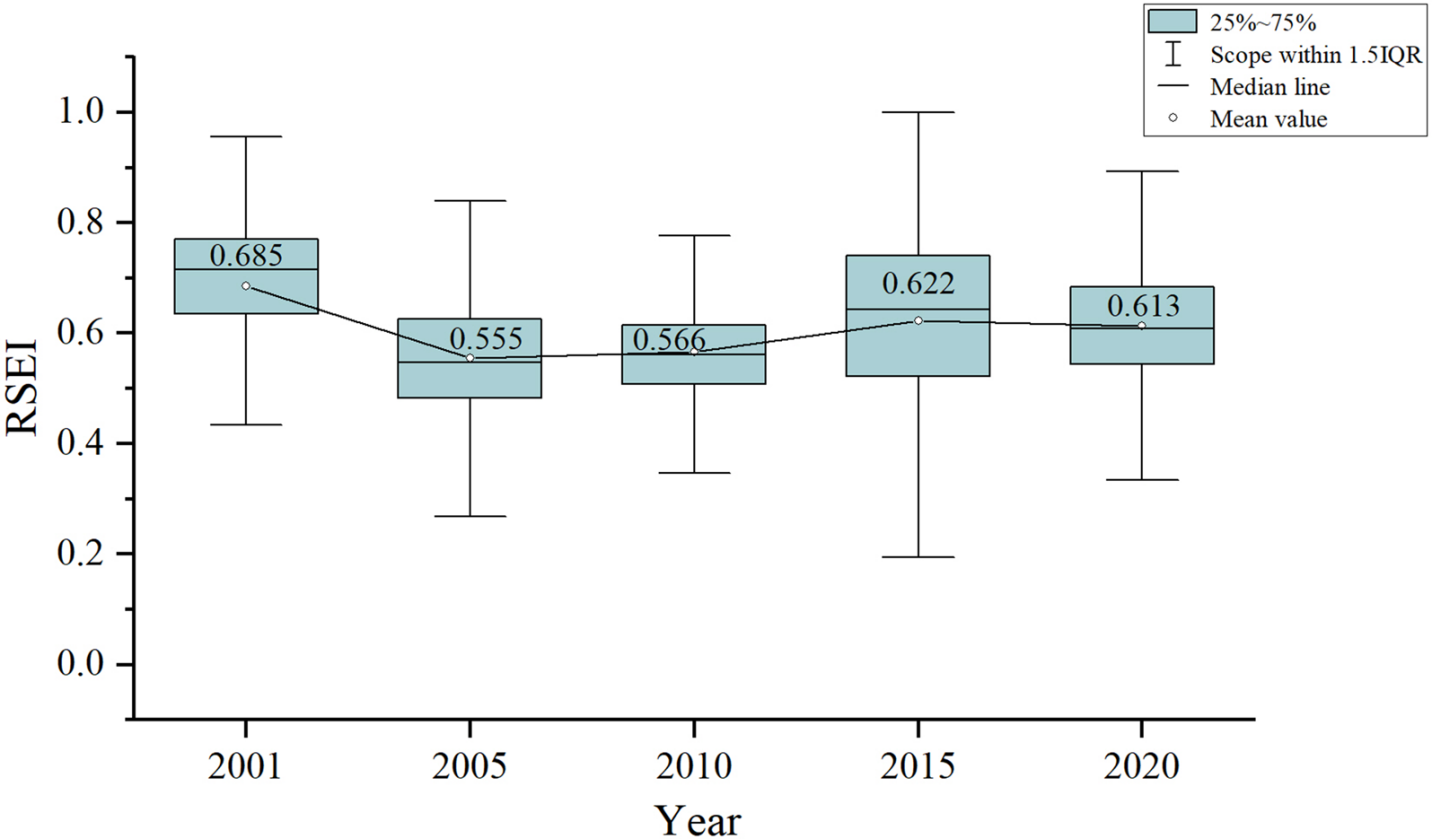
Broken line chart of average RSEI in northern Anhui from 2001 to 2020.

In this paper, PCA was used to couple the four indicators for the five years of 2001, 2005, 2010, 2015, and 2020 in northern Anhui to construct the RSEI. The average value of the RSEI and its change range in each year were also calculated (Fig. 3). As shown in Fig. 3, the RSEI in 2010 was relatively concentrated, the RSEI in 2015 was the most scattered, and the RSEI values in the other years ranged between the two. The results showed that the mean value of the RSEI in 2001 was 0.685, and this was the highest value among the 5 years analyzed in this paper. The mean values of the RSEI in 2005 and 2010 changed only slightly, being 0.555 and 0.566, respectively. In 2015, the mean value of the RSEI increased to 0.622, while by 2020, the average RSEI had fallen slightly to 0.613. On the whole, the RSEI showed a rapid decline from 2001 to 2005, but the decline rate slowed down from 2005 to 2020 and began to show a trend of improvement.

To further quantitatively analyze the RSEI, the ecological environment quality was classified according to an interval of 0.2 and divided into five grades: poor, fair, moderate, good, and excellent^32^. The area and proportion of each ecological grade in northern Anhui over 5 years were also counted. According to the statistics shown in Table 4, in 2001, the proportion of the area when the RSEI grade was poor or fair was 4.12%, and 14.88% of the area had a moderate grade classification; the largest proportion of the area (81%) had an RSEI grade of good or excellent. The data obtained in 2005 and 2010 yielded similar results. The proportions of the RSEI grade when the area was poor or fair were 8.08% and 5.22% in 2005 and 2010, respectively, which were increases compared with the proportion of the same grade in 2001. The proportion of the area with a moderate RSEI grade increased greatly, reaching 59.93% and 63.43% in 2005 and 2010, respectively. When the RSEI grade was good or excellent, the proportion of the area was 31.99% and 31.35% in 2005 and 2010, respectively. Compared with the same period in 2001, the area ratio of good and excellent RSEI grades dropped significantly, indicating that the ecological environment quality exhibited a downward trend in 2005 and 2010 when compared with 2001. In 2015, when the RSEI grade was poor or fair, the area ratio changed little compared with the same grade in the previous period, which was 8.94%. For the areas with an RSEI grade of moderate, the area ratio decreased compared with the previous period, and for RSEI grades of excellent or good, the area ratio increased, reaching 31.16% and 59.9%, respectively, indicating that the environmental conditions had improved in 2015. By 2020, the area ratio of RSEI grades with poor and fair grades along with good and excellent grades decreased compared with the previous period, accounting for 4.2% and 53.03% of the total area, respectively. At the moderate level, the area ratio increased slightly compared with the same period in 2015, to 42.77%, and the overall ecological environment quality decreased slightly when compared with 2015.

**Table 4:** Percentage of RSEI grade in northern Anhui from 2001 to 2020.

Grade	Year				
	2001 (%)	2005 (%)	2010 (%)	2015 (%)	2020 (%)
Poor (0.0–0.2)	0.38	0.29	0.39	0.78	0.39
Fair (0.2–0.4)	3.74	7.79	4.83	8.16	3.81
Moderate (0.4–0.6)	14.88	59.93	63.43	31.16	42.77
Good (0.6–0.8)	68.41	29.46	27.96	49.21	46.13
Excellent (0.8–1.0)	12.59	2.53	3.39	10.69	6.9

**Figure 4:**
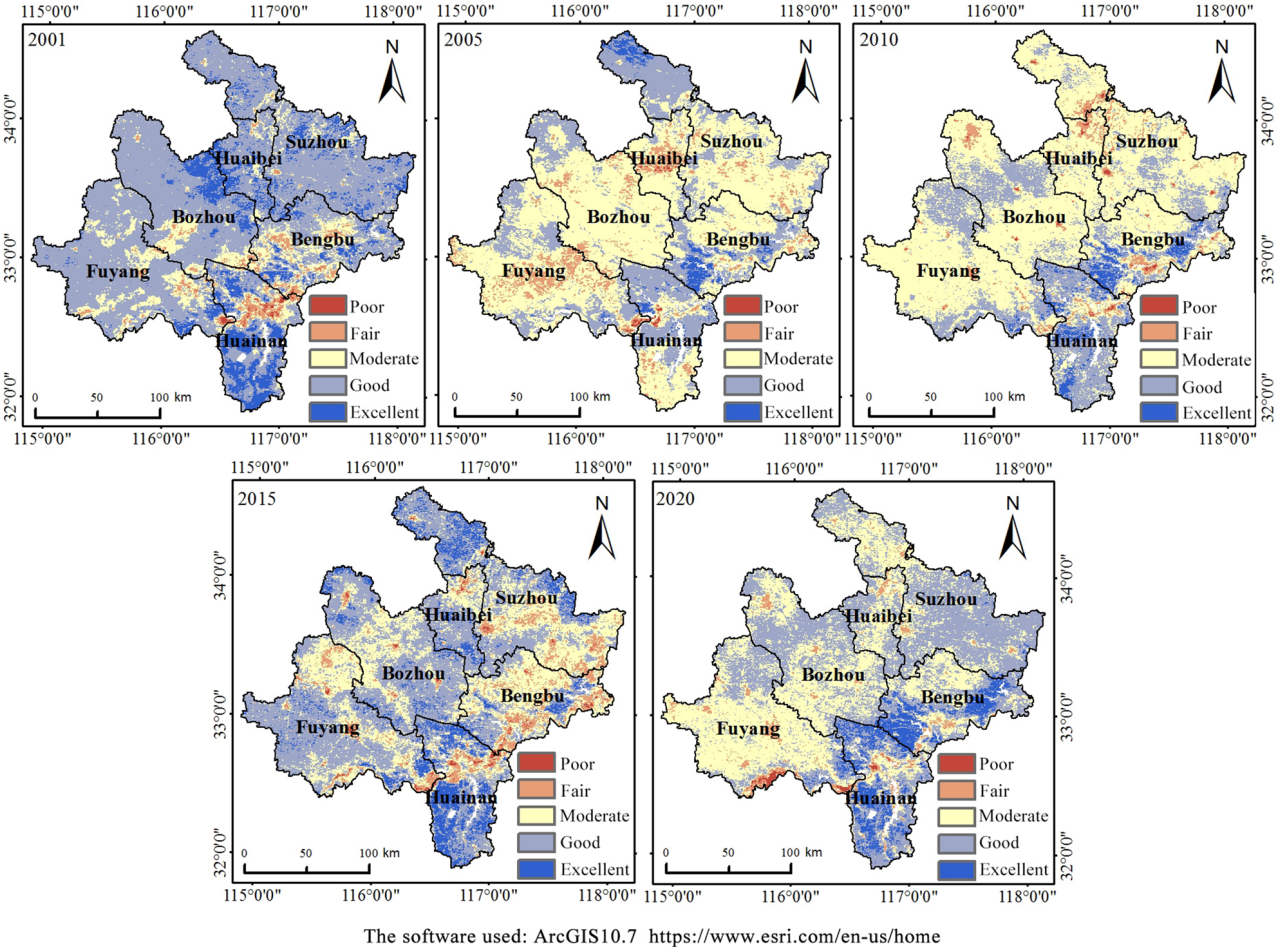
RSEI grade map of northern Anhui from 2001 to 2020.

To better analyze the spatial differentiation characteristics, the RSEI grade distribution ratios of six cities in northern Anhui were calculated (Table 5). Combined with the data shown in Fig. 4, it was clearly demonstrated that Huainan in the southern part of northern Anhui had a better and more stable ecological environment quality than the other cities from 2001 to 2020. A relatively high proportion of the areas had a good or excellent RSEI. Except in 2005, this proportion exceeded 70% in all other years, with values of 76.15% (2001), 52.98% (2005), 75.37% (2010), 76.06% (2015), and 77.73% (2020). From 2001 to 2020, the ecological environment quality of Bengbu and Suzhou showed a trend of an initial decline, followed by an increase. When the RSEI was good or excellent, the area ratio reached the lowest in 2015 (32.88%) and 2010 (17.56%). The ecological environment quality of the other three cities, Huaibei, Fuyang, and Bozhou, showed a trend of declining fluctuations over time.

**Table 5:** Statistics on the proportion of RSEI grades of cities in northern Anhui from 2001 to 2020.

Year	Grade	Bengbu (%)	Huainan (%)	Huaibei (%)	Fuyang (%)	Suzhou (%)	Bozhou (%)
2001	Poor	0.34	1.92	0.00	0.37	0.01	0.05
	Fair	8.78	9.21	1.09	3.01	0.68	2.23
	Moderate	29.78	12.72	10.03	18.88	7.13	12.05
	Good	51.79	43.66	67.75	74.85	79.98	74.35
	Excellent	9.31	32.49	21.13	2.89	12.20	11.32
2005	Poor	0.03	0.61	1.62	0.45	0.00	0.01
	Fair	3.73	5.79	18.95	13.32	4.10	6.04
	Moderate	53.73	40.62	66.34	61.43	55.13	78.15
	Good	33.41	49.84	13.09	24.41	37.29	15.80
	Excellent	9.10	3.14	0.00	0.39	3.48	0.00
2010	Poor	0.55	0.48	0.71	0.18	0.52	0.23
	Fair	6.03	4.15	11.89	2.70	6.26	3.17
	Moderate	52.66	20.00	79.24	69.10	75.66	70.88
	Good	29.22	62.56	8.16	27.01	17.47	25.72
	Excellent	11.54	12.81	0.00	1.01	0.09	0.00
2015	Poor	1.40	1.65	0.64	0.83	0.35	0.27
	Fair	17.93	8.05	4.95	7.02	9.36	2.48
	Moderate	47.79	14.24	25.51	31.02	34.31	29.18
	Good	27.98	43.48	60.68	55.70	44.24	61.68
	Excellent	4.90	32.58	8.22	5.43	11.74	6.39
2020	Poor	0.00	0.54	0.01	1.37	0.00	0.00
	Fair	2.84	5.57	4.78	6.42	1.77	2.05
	Moderate	34.62	16.16	46.12	65.01	32.47	49.88
	Good	41.08	50.05	49.06	25.71	65.09	47.81
	Excellent	21.46	27.68	0.03	1.49	0.67	0.26

### Changes in the ecological environment quality in northern Anhui

**Figure 5:**
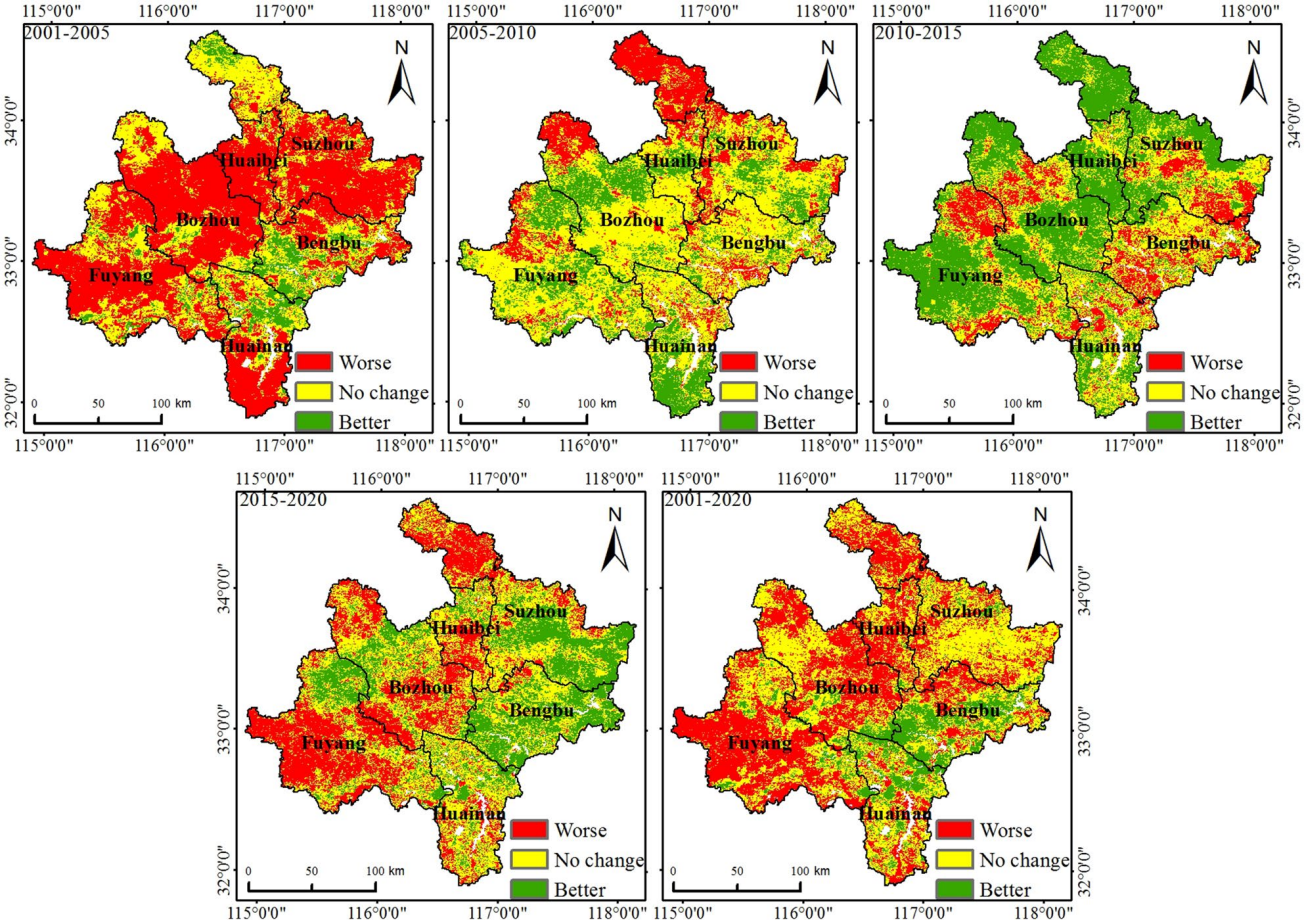
Change detection diagram of RSEI in northern Anhui.

**Figure 6:**
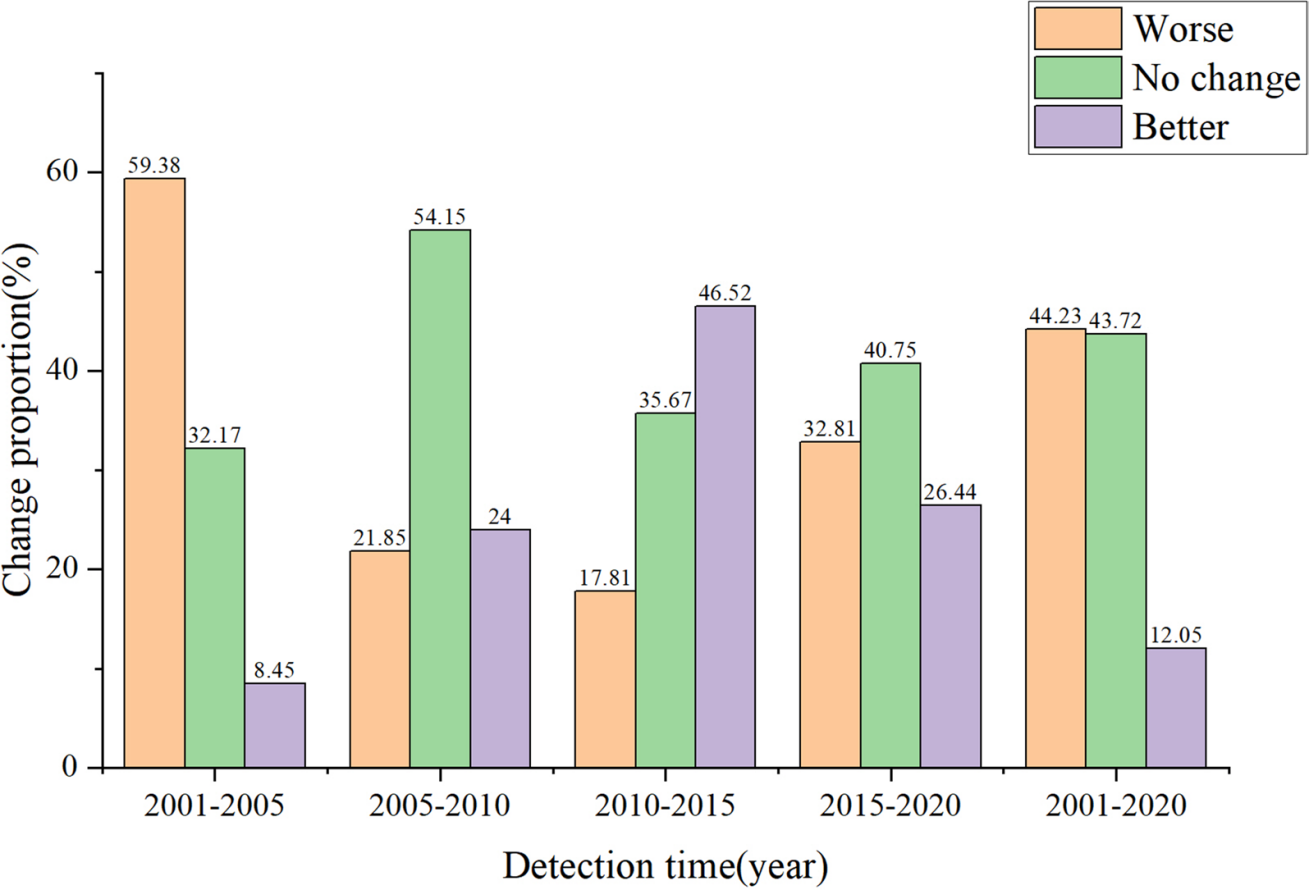
Scale map of RSEI change detection in northern Anhui.

Based on the above-mentioned classification of ecological environment quality, the difference detection and analysis of the changes in the ecological environment quality in 2001–2005, 2005–2010, 2010–2015, 2015–2020, and 2001–2020 were carried out (Figs. 5 and 6). From 2001 to 2005, the ecological environment quality of 59.38% of the various regions weakened; these areas were more widely distributed in Huaibei, Suzhou, Bozhou, and Fuyang. The areas with improved and unchanged ecological environment quality accounted for 8.45% and 32.17%, respectively; these areas had a relatively scattered spatial distribution. From 2005 to 2010, 21.85% of the areas had weakened ecological environment quality. These areas were mainly located in Bozhou and northern Suzhou. Areas with improved and unchanged ecological environment quality in this period accounted for 24% and 54.15% of northern Anhui, respectively, and were widely distributed in most areas of the six cities. From 2010 to 2015, the quality of 17.81% of the regional ecological environment declined, while areas with improved and unchanged regional ecological environment quality accounted for 46.52% and 35.67% of the study area, respectively. These areas were distributed to different degrees in each city, among which they were more widely distributed in Suzhou, Huaibei, Bozhou, and Fuyang. From 2015 to 2020, the areas where the quality had declined accounted for 32.81% of northern Anhui, while the areas with increased and unchanged ecological environment quality accounted for 26.44% and 40.75%, respectively. These areas were more widely distributed in Huainan, Bengbu, and Suzhou. According to the change detection results from 2001 to 2020, 44.23% of the regional ecological environment quality had declined, and the ecological environment quality in Fuyang, Bozhou, and Huaibei had been significantly degraded, while regions such as Huainan, Bengbu, and Suzhou also experienced different degrees of degradation.

### Detection of influencing factors of ecological environment quality

To further explore the driving factors of the ecological environment quality in northern Anhui, we divided the rainfall, temperature, vegetation coverage, land use, nighttime light, and population density in 2001, 2005, 2010, 2015, and 2020 into social and natural factors as independent variables Xi (Table 6) based on previous studies^53,54^ and data availability, and the RSEI value was set as the dependent variable *Y*. A grid of 1200 m × 1200 m was set in ArcGIS, and the values corresponding to the points in the grid were extracted to the table. After removing the abnormal values, a total of 28,854 sampling points were obtained. Finally, the data were run in Geodetector to explore the influence of each factor on the spatial differentiation characteristics of the RSEI. To minimize the average dispersion variance within the layer and maximize the average dispersion variance between the layers, the natural breakpoint method was used to divide the rainfall, temperature, vegetation coverage, nighttime light, and population density into 11 kinds of data, while the land use was classified according to the annual International Geosphere-Biosphere Programme (IGBP).

**Table 6:** Influencing factors.

Type	Influencing factors	
Social factors	X1	Rainfall
	X2	Temperature
	X3	Vegetation coverage
Natural factors	X4	Land use
	X5	Nighttime light
	X6	Population density

As shown in Table 7, the order of the average *q* values corresponding to each detection factor was vegetation coverage > rainfall > nighttime light > land use > temperature > population density. From the perspective of the explanatory power of influencing factors, vegetation coverage had the strongest explanatory power for the spatial differentiation characteristics of the RSEI, reaching 28.9%, making it the main factor influencing the RSEI. The explanatory powers of rainfall also reached over 10%, making rainfall the secondary factor influencing RSEI. The explanatory powers of temperature, land use, nighttime light, and population density for the spatial differentiation characteristics of the RSEI were all weak at lower than 10%. In general, natural factors had a greater impact on the RSEI than social factors.

**Table 7:** Results of factor detection.

Year	*P* and *q*	X1	X2	X3	X4	X5	X6
2001	*q*	0.150	0.021	0.540	0.075	0.072	0.055
	*p*	0.000	0.000	0.000	0.000	0.000	0.000
2005	*q*	0.167	0.087	0.008	0.012	0.009	0.006
	*p*	0.000	0.000	0.000	0.000	0.000	0.002
2010	*q*	0.129	0.061	0.180	0.104	0.143	0.115
	*p*	0.000	0.000	0.000	0.000	0.000	0.000
2015	*q*	0.085	0.064	0.350	0.085	0.118	0.072
	*p*	0.000	0.000	0.000	0.000	0.000	0.000
2020	*q*	0.071	0.114	0.365	0.079	0.117	0.089
	*p*	0.000	0.000	0.000	0.000	0.000	0.000
–	Average *q*	0.120	0.069	0.289	0.071	0.092	0.067

The results of interactive detection showed that, compared with a single factor, the effects of all the detection factors on the spatial differentiation characteristics of the RSEI in northern Anhui were synergistically enhanced, and the effects of all factors influencing the RSEI were not independent (Table 8). The nonlinear enhancement mode and the two-factor enhancement mode appeared about the same number of times. Among them, the non-linear enhancement mode appeared 38 times, while the two-factor enhancement mode appeared 37 times. From the perspective of the interactive explanatory power of pairs of detection factors, the interaction between vegetation coverage and other factors had a strong explanatory power for the spatial differentiation characteristics of the RSEI in northern Anhui. Among them, the interaction between vegetation coverage and rainfall (average *q* = 0.404) was the most obvious, followed by vegetation coverage and temperature (average *q* = 0.359), along with vegetation coverage and nighttime light (average *q* = 0.312). The least obvious interactions were between land use and population density (average *q* = 0.102), along with nighttime light and population density (average *q* = 0. 109), both of which had an explanatory power below 11%.

**Table 8:** Results of interactive detection.

Combination of factors	2001	2005	2010	2015	2020	Average
X1 ∩ X2	0.223^a^	0.268^a^	0.245^a^	0.231^a^	0.26^a^	0.245
X1 ∩ X3	0.58^b^	0.203^a^	0.353^a^	0.445^a^	0.437^a^	0.404
X1 ∩ X4	0.225^a^	0.182^a^	0.239^a^	0.18^a^	0.147^b^	0.195
X1 ∩ X5	0.238^a^	0.197^a^	0.275^a^	0.219^a^	0.177^b^	0.221
X1 ∩ X6	0.213^a^	0.186^a^	0.233^b^	0.177^a^	0.167^a^	0.195
X2 ∩ X3	0.55^b^	0.127^a^	0.248^a^	0.414^a^	0.455^b^	0.359
X2 ∩ X4	0.108^a^	0.107^a^	0.163^b^	0.159^a^	0.191^b^	0.146
X2 ∩ X5	0.109^a^	0.114^a^	0.202^b^	0.194^a^	0.217^b^	0.167
X2 ∩ X6	0.091^a^	0.113^a^	0.166^b^	0.156^a^	0.18^b^	0.141
X3 ∩ X4	0.547^b^	0.027^a^	0.218^b^	0.362^b^	0.376^b^	0.306
X3 ∩ X5	0.549^b^	0.025^a^	0.237^b^	0.372^b^	0.375^b^	0.312
X3 ∩ X6	0.548^b^	0.021^a^	0.221^b^	0.368^b^	0.377^b^	0.307
X4 ∩ X5	0.106^b^	0.022^a^	0.173^b^	0.137^b^	0.133^b^	0.114
X4 ∩ X6	0.099^b^	0.018^a^	0.158^b^	0.111^b^	0.126^b^	0.102
X5 ∩ X6	0.085^b^	0.014^b^	0.17^b^	0.131^b^	0.146^b^	0.109

^a^Non-linear enhancement relationship.

^b^Two-factor enhancement relationship.

The results of ecological detection showed that the proportion of the combination of factors with no significant difference in the RSEI changes in northern Anhui was about twice that of the combination of factors with significant differences (Table 9). For example, the ecological detection results of 51 pairs of interaction factors, such as vegetation coverage and land use, vegetation coverage and nighttime light, vegetation coverage and population density, land use and population density, and nighttime light and population density, were *N*, indicating that these combinations did not have significant differences in the impact of the spatial differentiation characteristics of the RSEI. The results of the other 24 pairs of interacting factors were *Y*, indicating that these combinations had significant differences in the impact of the spatial differentiation characteristics of the RSEI.

**Table 9:** Results of ecological detection.

Combination of factors	2001	2005	2010	2015	2020
X1 ∩ X2	N	N	N	N	Y
X1 ∩ X3	Y	N	Y	Y	Y
X1 ∩ X4	N	N	N	N	N
X1 ∩ X5	N	N	Y	Y	Y
X1 ∩ X6	N	N	N	N	Y
X2 ∩ X3	Y	N	Y	Y	Y
X2 ∩ X4	Y	N	Y	Y	N
X2 ∩ X5	Y	N	Y	Y	N
X2 ∩ X6	Y	N	Y	N	N
X3 ∩ X4	N	N	N	N	N
X3 ∩ X5	N	N	N	N	N
X3 ∩ X6	N	N	N	N	N
X4 ∩ X5	N	N	Y	Y	Y
X4 ∩ X6	N	N	N	N	N
X5 ∩ X6	N	N	N	N	N

Y indicates that there is a significant difference between the two factors on RSEI; N indicates no significant difference.

**Figure 7:**
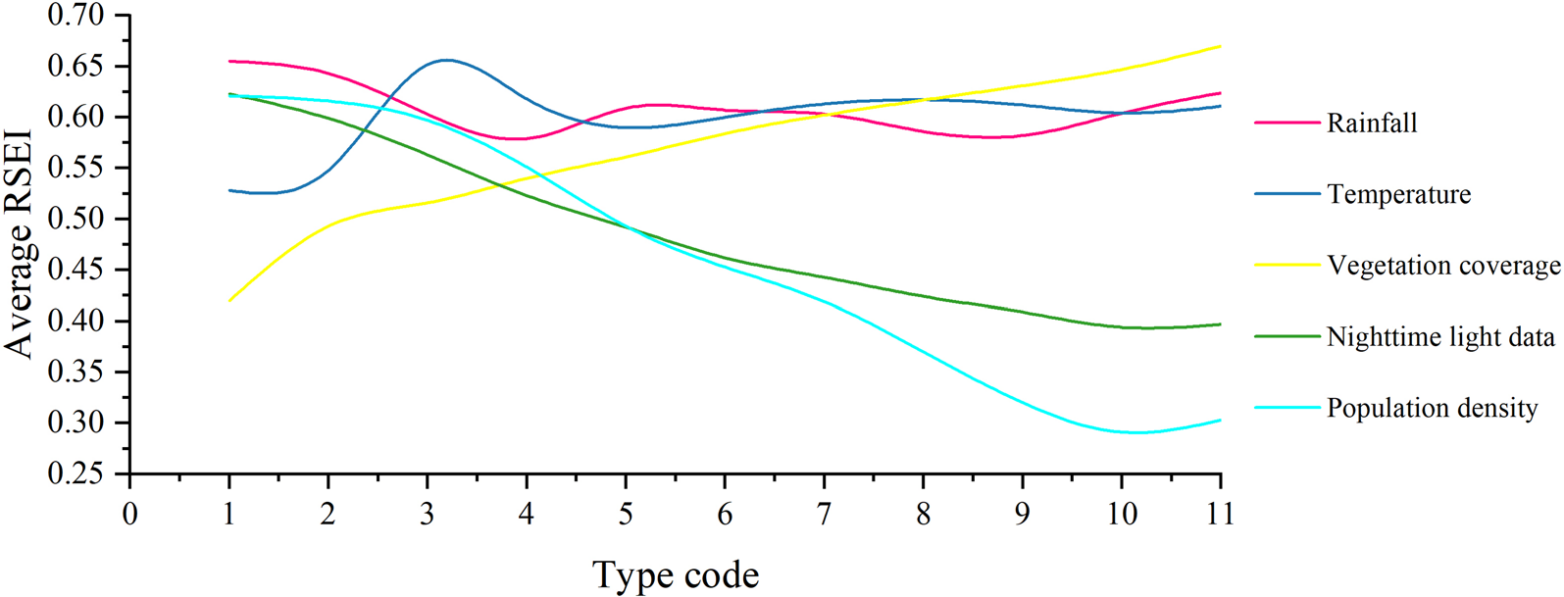
Change trend between average RSEI and influencing factors.

Based on an analysis of risk detection, Table 10 shows the RSEI values corresponding to the type code for each influencing factor, except for land use; because there are many types of land use and not every type is included in the study area, the RSEI corresponding to the land use is shown in Table 11 separately. The results passed the statistical test at 95% confidence level. Figure 7 illustrates how the RSEI changes as the impact factors increase. The type codes on the horizontal axis were codes that reclassified the values of influencing factors in each grid from small to large according to the natural breakpoint method, and each code represented a numerical interval. As shown in Table 10, each column represented the average value of the RSEI corresponding to the influencing factor in a certain numerical interval. The average value of the RSEI of each influencing factor in the corresponding numerical interval in the 5 years was further calculated (Fig. 7). The natural breakpoint method can maximize the classification of similar values into one level, so the average value of RSEI corresponding to the type code 0–11 can reflect the effect of influence factors of different sizes on RSEI. The results showed that with the increase in vegetation coverage, the RSEI showed a trend of increasing, and the highest average RSEI was 0.670. As the nighttime light and population density increased, the RSEI showed a trend of decreasing, and the highest average RSEI values were 0.623 and 0.621, respectively. Rainfall and temperature had no obvious trend effects on RSEI. The temperature reached the maximum at code 3, the rainfall reached the maximum at code 1, and the highest average RSEI values were 0.655 and 0.652, respectively. Among all land use types, permanent wetlands had the largest average RSEI.

**Table 10:** RSEI corresponds to the type codes of X1, X2, X3, X5, and X6 (confidence level 95%).

Year	Factors	Type code										
		1	2	3	4	5	6	7	8	9	10	11
2001	X1	0.656	0.730	0.639	0.595	0.677	0.708	0.735	0.743	0.715	0.706	0.726
	X2	0.491	0.681	0.714	0.673	0.685	0.703	0.691	0.659	0.697	0.680	0.718
	X3	0.407	0.495	0.541	0.581	0.623	0.662	0.694	0.716	0.734	0.748	0.765
	X5	0.694	0.696	0.653	0.615	0.562	0.534	0.489	0.480	0.401	0.384	0.359
	X6	0.691	0.697	0.678	0.615	0.538	0.487	0.475	0.359	0.314	0.254	0.280
2005	X1	0.646	0.628	0.572	0.528	0.520	0.495	0.501	0.524	0.538	0.568	0.540
	X2	0.601	0.574	0.774	0.699	0.602	0.570	0.527	0.535	0.557	0.546	0.547
	X3	0.497	0.537	0.551	0.566	0.559	0.561	0.558	0.557	0.557	0.562	0.538
	X5	0.545	0.560	0.561	0.548	0.541	0.528	0.518	0.491	0.456	0.461	0.450
	X6	0.552	0.557	0.560	0.546	0.522	0.504	0.474	0.456	0.422	0.377	0.387
2010	X1	0.644	0.590	0.573	0.550	0.536	0.536	0.552	0.515	0.522	0.506	0.527
	X2	0.385	0.350	0.530	0.481	0.507	0.530	0.576	0.587	0.573	0.588	0.554
	X3	0.403	0.493	0.506	0.525	0.542	0.553	0.562	0.567	0.572	0.588	0.627
	X5	0.601	0.562	0.551	0.544	0.527	0.518	0.503	0.475	0.420	0.372	0.273
	X6	0.590	0.566	0.542	0.495	0.436	0.403	0.327	0.334	0.252	0.219	0.219
2015	X1	0.738	0.693	0.619	0.604	0.652	0.636	0.610	0.575	0.549	0.638	0.741
	X2	0.578	0.547	0.682	0.662	0.591	0.655	0.659	0.643	0.599	0.559	0.606
	X3	0.393	0.467	0.479	0.511	0.537	0.580	0.614	0.647	0.674	0.700	0.728
	X5	0.646	0.605	0.544	0.465	0.428	0.350	0.325	0.282	0.257	0.354	0.637
	X6	0.629	0.644	0.620	0.549	0.454	0.401	0.368	0.297	0.247	0.241	0.241
2020	X1	0.590	0.575	0.614	0.620	0.660	0.662	0.616	0.572	0.584	0.601	0.584
	X2	0.586	0.588	0.562	0.575	0.564	0.542	0.613	0.663	0.634	0.645	0.628
	X3	0.402	0.474	0.502	0.519	0.544	0.564	0.580	0.596	0.617	0.639	0.694
	X5	0.631	0.573	0.507	0.441	0.400	0.382	0.380	0.394	0.512	0.398	0.266
	X6	0.643	0.614	0.586	0.550	0.515	0.469	0.450	0.406	0.364	0.363	0.388
Average	X1	0.655	0.643	0.603	0.579	0.609	0.607	0.603	0.586	0.582	0.604	0.624
	X2	0.528	0.548	0.652	0.618	0.590	0.600	0.613	0.617	0.612	0.604	0.611
	X3	0.420	0.493	0.516	0.540	0.561	0.584	0.602	0.617	0.631	0.647	0.670
	X5	0.623	0.599	0.563	0.523	0.492	0.462	0.443	0.424	0.409	0.394	0.397
	X6	0.621	0.616	0.597	0.551	0.493	0.453	0.419	0.370	0.320	0.291	0.303

**Table 11:** RSEI corresponds to the type codes of X4 (confidence level 95%).

Year	Type code								
	5	7	9	10	11	12	13	14	17
2001	–	–	0.566	0.560	0.596	0.695	0.548	0.637	0.580
2005	–	–	0.574	0.643	0.662	0.554	0.519	0.656	0.654
2010	0.497	–	0.462	0.604	0.693	0.573	0.407	0.514	0.618
2015	0.620	0.613	0.492	0.545	0.612	0.637	0.436	0.574	0.560
2020	0.680	0.501	0.495	0.575	0.690	0.624	0.488	0.577	0.644
Average	0.599	0.557	0.518	0.585	0.651	0.617	0.480	0.592	0.611

Code 5 represents mixed forests; code 7 represents open shrublands; code 9 represents savannas; code 10 represents grasslands; code 11 represents permanent wetlands; code 12 represents croplands; code 13 represents urban areas; code 14 represents cropland—natural vegetation mosaic; code 17 represents water bodies.

## Discussion

### Application of ecological environment quality monitoring in the GEE

Rapid urbanization and development are causing increasingly severe pressure on the regional environment in northern Anhui. Paying attention to the need for regional ecological protection while achieving coordination between economic development and the environment has become an important issue that needs to be resolved in light of the desire to maintain high-speed and high-quality urbanization in northern Anhui. At present, the establishment of each index employed here is limited by image quality during research involving the use of an RSEI to monitor regional ecological environment quality. For example, the existence of areas with frequent cloud cover will result in missing data. It is usually necessary to use images from neighboring years to replace these missing data. As an alternative, research can be successfully conducted on a small scale^55^. The scope and scale of this type of research are limited in time and space based on certain restrictions related to accuracy and comparability. In recent years, the GEE platform has developed rapidly. This platform allows researchers to access, operate, and visualize massive amounts of remote sensing data by accessing Web API and a web-based interactive development environment at any time^56–59^. When calculating the RSEI in northern Anhui from 2001 to 2020, based on the powerful cloud computing capabilities of the GEE, this paper contributed to the field by establishing a time threshold to ensure the consistency of the time periods studied and used cloud removal and image median equalization to improve the original image quality. Compared with traditional methods, the result is a more authentic and objective calculation of the RSEI, which greatly improved the work efficiency^60,61^. In addition, based on the characteristics of the cloud platform, this method can be quickly applied to other regions and has higher application potential than traditional local computing.

### Influencing factors of ecological environment quality in northern Anhui

The risk detection results showed that different factors influenced the ecological environment quality in various ways. For example, with the increase of vegetation coverage, the RSEI showed an increasing trend, while with the increase of nighttime light and population density, the RSEI showed a decreasing trend, and the ecological environment quality of the wetland was the highest. This is consistent with the research of some scholars. For example, Li et al. found that the improvement of the ecological environment of the Grand Canal is closely related to the increase of vegetation coverage^62^; He et al. found that the increase of vegetation coverage is the main factor to improve the ecological environment of the scenic spot^63^; Zhang et al. found that urbanization has a negative impact on the ecological environment^64^. A high level of vegetation coverage can not only provide an ideal environment for animal and plant growth. It also has many functions, including purifying air, preventing water and soil loss, and reducing the heat island effect, which can greatly improve the ecological environment. Wetlands are the junction of land and water areas and have a diverse range of biological resources. They can not only change air humidity based on plant transpiration but also regulate water resources in rainy and dry climates. At the same time, wetlands can degrade pollutants by relying on soil microorganisms, improving the regional ecological environment quality. High population density and night light imply the presence of high-intensity human activities. In terms of land use, these activities tend to occupy the original natural environment and transform it into an artificial environment. At the same time, these activities continue to consume natural resources to attain population growth and economic development. In addition, these activities generate large amounts of waste gas and waste water, which worsens the environmental damage and pollution, negatively impacting the ecological environment. Moreover, the risk detection results showed that the RSEI fluctuated widely with increasing rainfall and temperature, but there were peaks in both. This indicates that although the relationship between temperature, rainfall, and RSEI is not very significant, it involves an optimal range of effects. Within this range, climatic conditions such as rainfall and temperature may have a more significant role in promoting plant growth, thus increasing the RSEI value. After exceeding this range, the promotion effect on plant growth weakens, causing RSEI to weaken.

The factor and interactive detection results showed that vegetation coverage and rainfall were the most important factors affecting the ecological environment quality in northern Anhui. After a synergistic effect, their contribution to this quality was significantly enhanced. This is mainly because this region is a transition zone from a warm temperate zone to a northern subtropical zone. Its annual rainfall is moderate, sunlight is sufficient, and the terrain is flat, making it suitable for vegetation growth. However, the regional water supply is mainly dependent on natural precipitation in the plain area. At the same time, the soil permeability and water retention are poor, making it extremely vulnerable to drought and flood. Therefore, rainfall has become the key influencing factor of regional environmental quality. It works in harmony with temperature, soil, light, and other factors to promote vegetation growth and improve regional ecological environment quality.

### Policy impact

**Figure 8:**
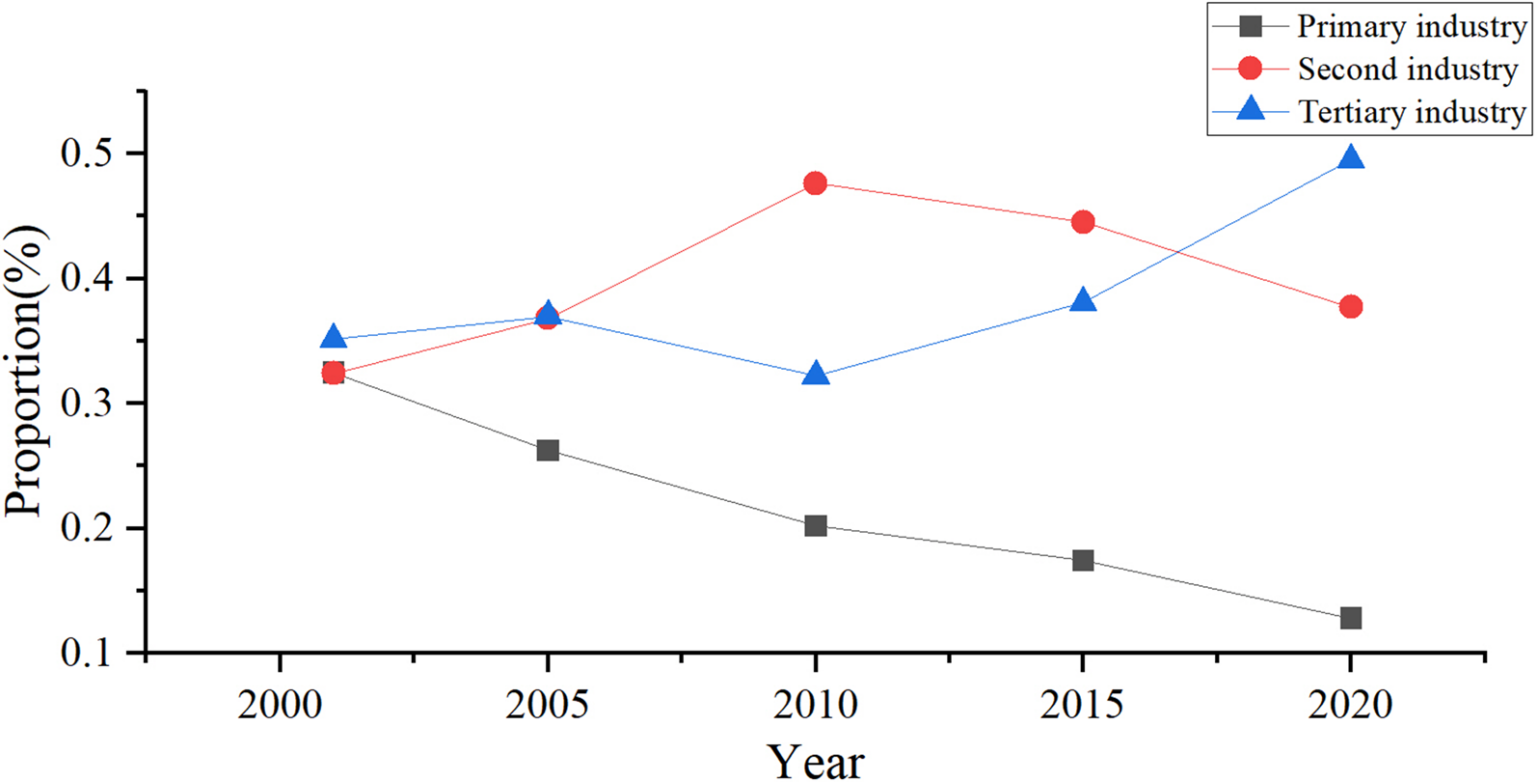
The proportion of the primary industry, secondary industry, and tertiary industry in regional GDP from 2001 to 2020.

Figure 8 depicts the changes in industrial structure in northern Anhui from 2001 to 2020. The proportion of the primary industry decreased year on year from 2001 to 2020. The proportion of the secondary industry continued to rise before 2010. However, after 2010, it turned into a continuous decline. The tertiary industry underwent a fluctuating decline before 2010 but continued to rise after 2010. After consulting a statistical yearbook and research literature, we found that before 2010, the economy of northern Anhui was mainly based on industrial development and coal mining. Many factories encroached on forests and grasslands. Mining led to increased destruction of vegetation, increased pollutants such as wastewater and waste materials, and increased land and river pollution, leading to a decline in its ecological environment quality. Since 2010, Anhui Province has issued *Opinions on Further Strengthening Land Conservation and Intensive Use* and *Opinions on Promoting Sustainable, Healthy, and Rapid Economic Development*. The government began to focus on applying new technologies, expanding its level of opening up, promoting regional industrial adjustments, and constantly increasing the proportion of tertiary industries in regional GDP. This caused the urban industrial economy to move towards an intensive development model, and also increased coordination between the urban ecological environment and economic development. Anhui Province has also actively reformed its household registration management system to enable its labor can flow to cities. The scale effect created by a concentrated population and its transformed structure has concentrated resource consumption and pollution emissions. This has concentrated the supply of multiple elements and facilities, reduced the per capita supply and governance costs, improving the urban ecological environment. At the same time, Anhui Province issued the *Twelfth Five Year Plan for the Renovation and Development of Noncoal Mines in Anhui Province*, the *Overall Plan of Anhui Province's Ten Million mu Forest Growth Project (2012–2016)*, and the *Implementation Opinions on Strengthening Key Environmental Protection Work*. The government has begun to actively implement measures including mine governance, water governance, the conversion of farmland to forests, and natural forest projects. These will help to regulate the climate, increase vegetation coverage, optimize forestry resource use, and curb the water and soil loss as well as natural disasters while improving the stability of the ecosystem and ecological service functions. This can enable the ecological environment of northern Anhui to improve year by year, which is consistent with the changing trend of ecological environment quality reflected in the model results.

### Limitations and prospects

The current research is not without limitations. First, although the RSEI model has been widely used, its indicator system still needs to be improved. For example, Wan et al. used PCA to couple indicators of greenness, humidity, dryness, heat, and PM_2.5_ (particulate matter with a diameter of less than 2.5 µm suspended in the air) concentration to construct a new RSEI to assess the ecological environment quality in Cangzhou^65^. Wang et al. created an improved RSEI model for arid areas based on green, humidity, salinity, heat, and land degradation, and conducted a spatio-temporal analysis of the environment of the Ulanbuh Desert in China^66^. In addition, this article is limited by the large scale of the land analyzed in northern Anhui. The type and accuracy of the data obtained are subject to certain restrictions. The selection of factors influencing the RSEI also needs to be enriched, and terrain, soil, road traffic, and other factors should be added in the future. In the future, when studying the factors driving the ecological environment quality in northern Anhui, the index system of the RSEI model should be improved, and index factors that conform to the environmental characteristics of northern Anhui should be added. Second, high-precision data should also be obtained, such as the introduction of higher resolution Landsat remote sensing images. In addition, using the GWR regression model or spatial autocorrelation model to further explore the impact mechanism of each factor on the spatial heterogeneity of the RSEI will be the focus of future research.

## Conclusions

Understanding the temporal and spatial variation characteristics of the ecological environment quality in northern Anhui and exploring the driving mechanism of its spatial distribution characteristics are important measures to support the formulation of environmental protection policies, as well as to rationally develop and utilize resources, which is conducive to achieving sustainable social development. This paper used GEE to construct RSEI to analyze the temporal and spatial characteristics of the ecological environment quality in northern Anhui from 2001 to 2020, used geographical detectors to explore the influencing factors of the ecological environment quality in northern Anhui, and drew the following conclusions.

The ecological environment quality in northern Anhui declined rapidly from 2001 to 2005, mainly because the area of construction land in northern Anhui increased greatly during this period, the urban construction process was accelerated, and the ecological environment was severely damaged. However, the decline slowed from 2005 to 2020 and began to show an improving trend, mainly due to the support of various national and local environmental policies. The ecological environment quality of Bengbu and Suzhou initially declined and then improved; the environmental quality of the other three cities, Huaibei, Fuyang, and Bozhou, showed a downward and fluctuating trend over time. In contrast, the ecological environment quality of Huainan City was better and more stable.

The explanatory power of each factor influencing the spatial differentiation of environmental quality in northern Anhui obviously varied. Vegetation coverage was the main factor, while rainfall was a secondary factor influencing RSEI. Temperature, nighttime light, land use, and population density had a weak influence on the spatial differentiation characteristics of RSEI. There was an interaction between the factors that impact the quality of the environment in northern Anhui, and both showed a non-linear enhancement and a two-factor enhancement relationship. Among them, the interaction between vegetation coverage and rainfall (*q* = 0.404) was the most obvious. In addition, we found that vegetation abundance had a positive impact on the ecological environment quality, while population density and urbanization had a negative impact on the ecological environment quality, and the ecological environment quality of wetlands in land use was the highest.

## Data Availability

All data generated or analyzed during this study are included in this published article.
